# The interleukin-33-mediated inhibition of expression of two key genes implicated in atherosclerosis in human macrophages requires MAP kinase, phosphoinositide 3-kinase and nuclear factor-κB signaling pathways

**DOI:** 10.1038/s41598-019-47620-8

**Published:** 2019-08-05

**Authors:** Melanie L. Buckley, Jessica O. Williams, Yee-Hung Chan, Lucia Laubertová, Hayley Gallagher, Joe W. E. Moss, Dipak P. Ramji

**Affiliations:** 1 Cardiff School of Biosciences, Cardiff University, Sir Martin Evans Building, Museum Avenue, Cardiff, CF10 3AX United Kingdom; 2 Institute of Medical Chemistry, Biochemistry and Clinical Biochemistry, Faculty of Medicine, Comenius University, Sasinkova 2, 813 72 Bratislava, Slovakia

**Keywords:** Interleukins, Molecular medicine

## Abstract

Atherosclerosis, a chronic inflammatory disorder of the walls of arteries, causes more deaths worldwide than any other disease. Cytokines, which are present at high levels in atherosclerotic plaques, play important roles in regulating the initiation and the progression of the disease. Previous studies using animal and cell culture model systems revealed protective, anti-atherogenic effects of the cytokine interleukin-33 (IL-33). The action of this cytokine involves both the induction and suppression of expression of many genes. Unfortunately, the signaling pathways that are responsible for the inhibition of gene expression by this cytokine are poorly understood. Further studies are required given the important roles of genes whose expression is inhibited by IL-33 in key cellular processes associated with atherosclerosis such as monocyte recruitment, foam cell formation and lipoprotein metabolism. We have investigated here the roles of various known IL-33 activated signaling pathways in such inhibitory actions using RNA interference-mediated knockdown assays and monocyte chemotactic protein-1 and intercellular adhesion molecule-1 as model genes. Key roles were identified for extracellular signal-regulated kinase-1/2, p38α kinase, c-Jun N-terminal kinase-1/2, phosphoinositide 3-kinase-γ, and p50 and p65 nuclear factor-κB in such inhibitory action of IL-33. These studies provide new insights on the signaling pathways through which IL-33 inhibits the macrophage expression of key atherosclerosis-associated genes.

## Introduction

Atherosclerosis, a major underlying cause of coronary heart disease (CHD), is associated with inflammation of the arterial wall^1,2^. The disease is initiated by endothelial cell dysfunction or activation in response to pro-inflammatory stimuli, such as modified low-density lipoproteins (LDL). This results in increased secretion of chemokines [e.g. monocyte chemotactic protein-1 (MCP-1)] by the endothelial cells along with the expression of cell surface adhesion molecules [e.g. intercellular adhesion molecule-1 (ICAM-1)], which together facilitate the recruitment, attachment and infiltration of circulating leukocytes into the subendothelial space^1,2^. Early atherosclerotic lesions are characterized by the accumulation of macrophages^1–3^. Lesion-resident macrophages take up LDL and oxidized LDL (oxLDL) through several processes, including macropinocytosis, phagocytosis and scavenger receptors-mediated endocytosis, thereby leading to their transformation into lipid-laden foam cells^1,2,4,5^. Furthermore, macrophages also serve as major sources of pro- and anti-inflammatory cytokines, which play crucial roles in the innate and adaptive immune responses associated with atherosclerosis^3,4,6,7^. Cytokines participate in all stages of the disease, including endothelial cell dysfunction, foam cell formation and secretion of matrix metalloproteinases involved in the degradation of extracellular matrix components^7,8^.

Many recent studies have demonstrated a protective anti-atherogenic role for the more recently identified cytokine interleukin (IL)-33^2,8^. Thus, reduced atherosclerosis was seen in the Apolipoprotein E deficient (ApoE^−/−^) mouse model system following administration of recombinant IL-33^9^. Several mechanisms were identified for the anti-atherogenic effects of IL-33: phenotypic switch from T helper (Th)1 to Th2 cells, increased production of anti-inflammatory cytokines IL-4 and IL-13, and the secretion of oxLDL antibodies^9^. Conversely, inhibition of IL-33 actions by the administration of a soluble decoy receptor promoted atherosclerosis development in this model system^9^. In addition, research in our laboratory demonstrated that IL-33 decreased foam cell formation *in vivo* in such ApoE^−/−^ mice^10^. Further studies on macrophages, which constitutively express the ST2 receptor^10–13^, *in vitro* revealed the potential mechanisms for the anti-foam cell action of IL-33. Thus, IL-33 caused a reduction in the uptake of modified LDL together with the expression of scavenger receptors SR-A, SR-B1 and CD36 (involved in the cellular uptake of modified LDL) and acyl-coA cholesterol acyltransferase-1 (involved in intracellular esterification of cholesterol)^10^. In contrast, cholesterol efflux from foam cells was stimulated by IL-33, and associated with up-regulated expression of cholesterol efflux transporters; ATP-binding cassette transporter (ABC)A1 and ABCG1^10^. The requirement of the ST2 receptor in the anti-foam cell actions of IL-33 together with associated changes in gene expression was confirmed by the use of bone marrow-derived macrophages (BMDM) from mice deficient in this receptor^10^. Subsequent studies have also confirmed the requirement of the ST2 receptor in the IL-33-mediated regulation of expression of other genes in human macrophages together with its anti-foam cell action^11,13^. IL-33 also inhibited macropinocytosis^5^, which contributes to the disease via the macrophage uptake of LDL particles^1^.

Both the up- and down-regulation of gene expression is associated with the cellular actions of IL-33. Several signaling pathways are activated by IL-33 in various cell types, including nuclear factor-κB (NF-κB), phosphoinositide 3-kinase (PI3K) and mitogen activated protein kinases (MAPK)^14–21^. Unfortunately, in contrast to activation, the signaling pathways involved in the suppression of gene expression by IL-33 are poorly understood mainly due to limited previous research and hence formed the focus of our current study on human macrophages using MCP-1 and ICAM-1 as model genes. We show key roles for extracellular signal-regulated kinase (ERK)-1/2, p38α MAPK, c-Jun N-terminal kinase (JNK)-1/2, PI3K-γ, and p50 and p65 NF-κB.

## Results

### The expression of MCP-1 and ICAM-1 genes in human macrophages is inhibited by IL-33

We have shown previously that IL-33 attenuates macrophage foam cell formation both *in vitro* and *in vivo*^10^. The action of IL-33 was conserved between human THP-1 macrophages, which have been used in numerous studies on macrophages in relation to atherosclerosis^22^, and primary cultures of human monocyte-derived macrophages (HMDM) and mouse BMDM, and also extended to the *in vivo* context^10^. These studies therefore validate the use of THP-1 macrophages and/or HMDM and/or BMDM for the investigation of IL-33 actions in relation to atherosclerosis^10^ as employed previously with other agents e.g.^23–25^.

**Figure 1:**
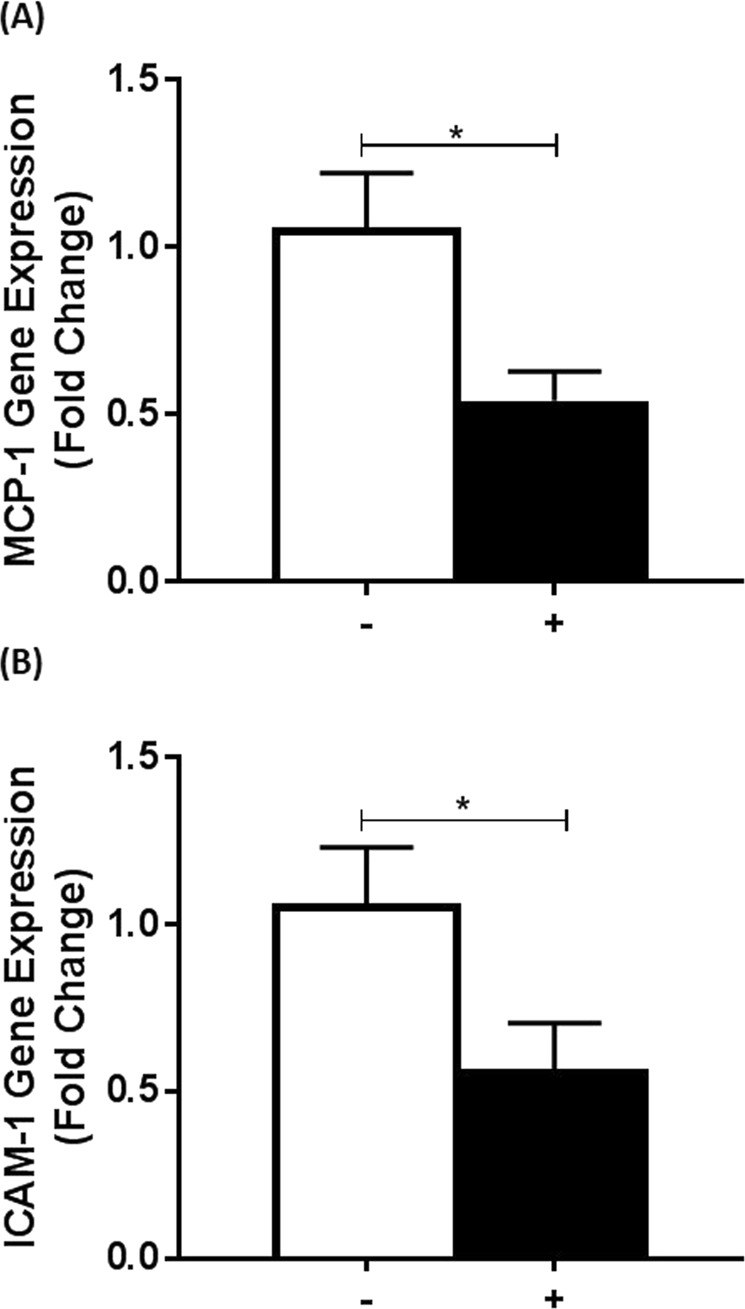
The effect of IL-33 on MCP-1 and ICAM-1 expression in primary macrophages. RT-qPCR for MCP-1 (**A**) and ICAM-1 (**B**) was carried out using cDNA against RNA from primary HMDMs that were incubated for 12 h in the presence of vehicle (−) or 25 ng/ml of IL-33 (+). The mRNA levels were calculated using the comparative Ct method and normalized to the housekeeping gene, GAPDH, with values from vehicle-treated control cells given an arbitrary value of 1. Data represents mean ± SEM from four independent experiments. Statistical analysis was carried out using an unpaired Student’s t test (*p ≤ 0.05).

We have previously shown that the expression of scavenger receptors SR-A1, SR-B1 and CD36 was attenuated by IL-33 in both THP-1 macrophages and HMDM^10^. Preliminary results showed that such an inhibitory action of IL-33 extended to other key atherosclerosis-associated genes: lipoprotein lipase, a key enzyme involved in the control of lipoprotein metabolism^26^; the adhesion protein ICAM-1, and the chemokines MCP-1, interferon gamma-induced protein-10 and macrophage inflammatory protein-1β in THP-1 macrophages (data not shown). Because of the crucial roles of MCP-1 and ICAM-1 in facilitating the recruitment and attachment of circulating leukocytes during the disease state^1,7,8^, subsequent studies focused on these atherosclerotic markers with a view of identifying the signaling pathways involved in the suppression of gene expression by the cytokine. Experiments were first carried out on primary cultures of HMDM to confirm that the inhibitory action of IL-33 on MCP-1 and ICAM-1 expression was not because of the use of the THP-1 cell line. As shown in Fig. 1, IL-33 significantly inhibited MCP-1 and ICAM-1 mRNA expression in HMDM (p = 0.040 and p = 0.011, respectively). The concentration of IL-33 used in these experiments (25 ng/ml) was within the physiological range (can reach up to 40 ng/ml)^10^.

Macrophages, including those derived from THP-1 monocytes, express the ST2 receptor^11,12,15,21^. For example, our previous studies showed that the ST2 receptor mRNA was expressed in both THP-1 macrophages and HMDM^10^ and this was extended in the current study to the protein level (Supplementary Fig. 1). Our previous research using BMDM from ST2 deficient mice showed that this receptor was required for all the IL-33-mediated changes in cellular processes and gene expression that we analyzed (e.g. inhibition of modified LDL uptake, cellular levels of total cholesterol and cholesteryl esters and the expression of SR-A1, CD36, SR-B1, ADAMTS-1 and ADAMTS-4; induction of cholesterol efflux and expression of ABCA1, ABCG1 and ApoE)^10,13^. Nevertheless, a neutralizing antibody with appropriate isotype control was used to investigate the requirement of the ST2 receptor for the changes in the expression of the MCP-1 and ICAM-1 genes by IL-33. Because of technical issues (i.e. compromised IL-33 response in the presence of isotype control antibody for MCP-1), conclusions could only be made for ICAM-1. The inhibition of ICAM-1 expression by IL-33 seen in THP-1 macrophages pre-incubated with the isotype control antibody was attenuated when the cells were instead pre-treated with anti-ST2 neutralizing antibody (Supplementary Fig. 2A). Despite the conservation of responses between THP-1 macrophages and HMDM, experiments were repeated in the latter cellular system. Although the decrease in ICAM-1 expression by IL-33 was not significant in HMDM pre-treated with the isotype control antibody, such a reduction was not seen following pre-treatment of the cells with ST2 neutralizing antibody (Supplementary Fig. 2B). This adds to our previously published studies that shows that the ST2 receptor is required for all the IL-33-mediated changes in cellular processes that we have investigated^10,13^.

### MAP kinases, NF-κB and PI3K-γ are involved in the IL-33-mediated down-regulation of MCP-1 and ICAM-1 expression

IL-33 activates multiple signaling pathways in several cellular systems^14–19^, including PI3K, NF-κB and the three MAPK cascades (ERK, JNK and p38), though very few studies have addressed their roles in relation to inhibition of gene expression by the cytokine. The requirement of key components in the MAPK, NF-κB and PI3Kγ signaling pathways in the inhibition of MCP-1 and ICAM-1 expression by IL-33 was therefore investigated by knockdown assays in THP-1 macrophages. Knockdown of targets was achieved using adenoviral encoding small hairpin RNAs (shRNAs) and/or small interfering RNAs (siRNAs).

**Figure 2:**
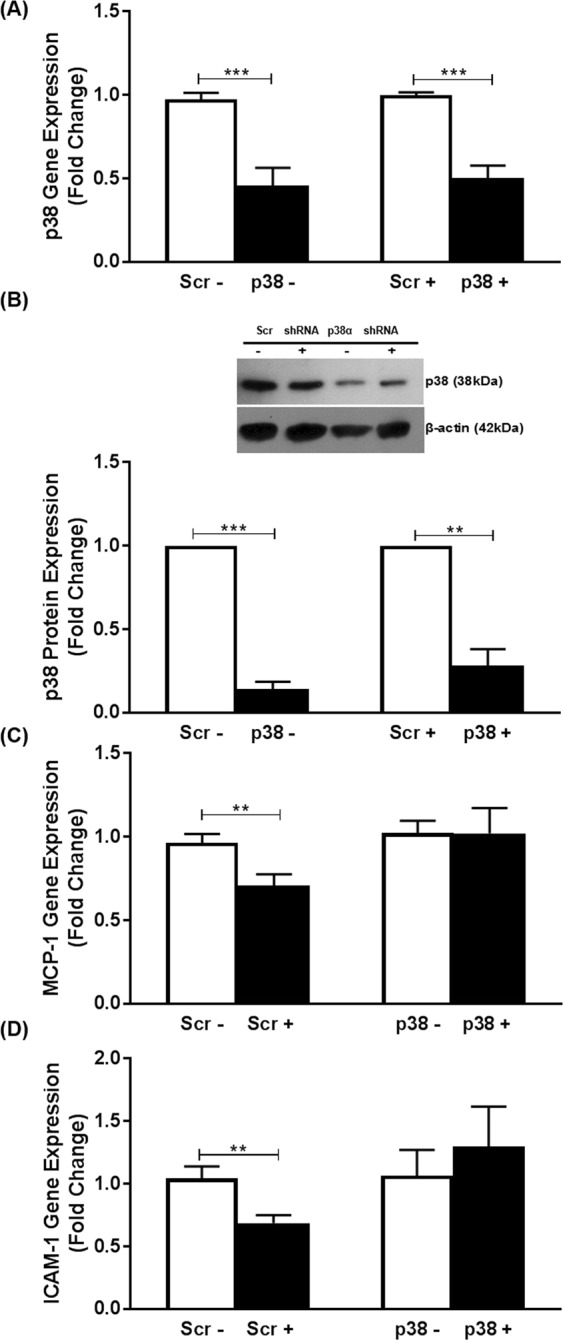
p38α is required for the IL-33-mediated inhibition of MCP-1 and ICAM-1 expression in human macrophages. Knockdown using adenovirus-encoding shRNA against p38α (p38) or scramble (Scr) sequence was carried out as Materials and Methods. THP-1 macrophages were then incubated for 12 h in the presence of vehicle (−) or 25 ng/ml IL-33 (+). The mRNA expression of p38α (**A**), MCP-1 (**C**) and ICAM-1 (**D**) was analyzed by RT-qPCR. Data represents mean ± SEM from six independent experiments. The expression of p38 protein levels was determined by Western blot analysis using β-actin as control (**B**). A representative image with signal from immunoreactive p38 or β-actin is shown (see Supplementary Fig. 4 for corresponding full-length image) with the histogram below it indicating p38 protein expression (mean ± SEM) normalized to β-actin from three independent experiments. The knockdown of p38α in vehicle- or IL-33 treated cells was determined in relation to the scramble control, which was arbitrarily assigned as 1 (**A**,**B**). The IL-33-mediated changes in MCP-1 and ICAM-1 expression in the scramble control was compared to that following knockdown of p38α (**C**,**D**) with values from cells infected with scramble shRNA or p38α shRNA and then treated with vehicle alone given an arbitrary value of 1. Statistical analysis was carried out using an unpaired Student’s t-test (**A**–**C**) or Man Whitney U test (**D**) (**p ≤ 0.01, ***p ≤ 0.001).

The use of shRNA targeting the major p38 isoform, p38α, produced a significant reduction in p38α mRNA and protein levels in vehicle and IL-33 treated cells respectively [55% (p < 0.001) and 50% (p < 0.001) respectively at mRNA expression and 86% (p < 0.001) and 72% (p = 0.002) respectively at the protein level] (Fig. 2A,B). RT-qPCR showed that the significant decrease in MCP-1 and ICAM-1 expression by IL-33 seen in cells transfected with the scramble control (p = 0.008 and p = 0.006, respectively) was attenuated following knockdown of p38α (Fig. 2C,D), thereby indicating a requirement for this kinase in the inhibitory action of this cytokine.

**Figure 3:**
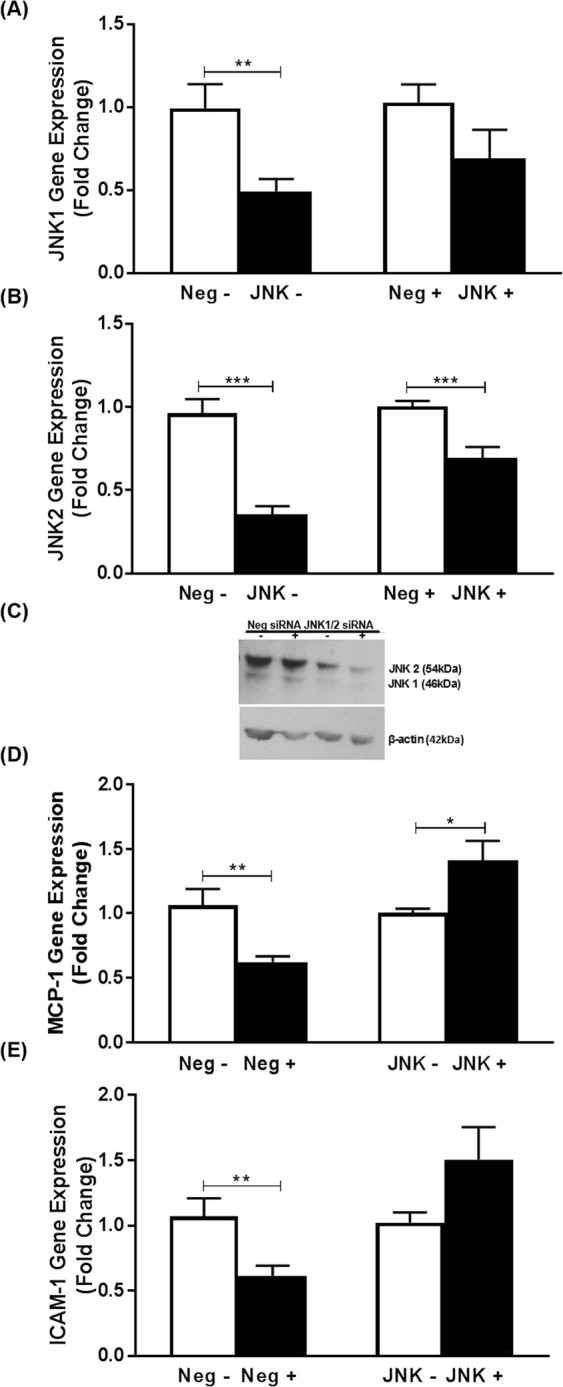
JNK1/2 is required for the IL-33-mediated inhibition of MCP-1 and ICAM-1 expression in human macrophages. Knockdown using siRNA against JNK1/2 (JNK) or negative control sequence (Neg) was carried out as Materials and Methods. THP-1 macrophages were then incubated for 12 h in the presence of vehicle (−) or 25 ng/ml IL-33 (+). The mRNA expression of JNK1 (**A**), JNK2 (**B**), MCP-1 (**D**) and ICAM-1 (**E**) was analyzed by RT-qPCR. Data represents mean ± SEM from five independent experiments. The knockdown of JNK1 (**A**) or JNK2 (**B**) in vehicle- or IL-33 treated cells was determined in relation to the negative control, which was arbitrarily assigned as 1 (**A**,**B**). The IL-33-mediated changes in MCP-1 and ICAM-1 expression in the negative control was compared to that following knockdown of JNK1/2 (**D**,**E**) with values from cells transfected with negative control siRNA or JNK1/2 siRNA and then treated with vehicle alone given an arbitrary value of 1. Statistical analysis was carried out using an unpaired Student’s t-test (*p ≤ 0.05; **p ≤ 0.01, ***p ≤ 0.001). The expression of JNK1/2 protein levels was determined by Western blot analysis using β-actin as a control (**C**). A representative image from two independent experiments with signals from immunoreactive JNK1/2 or β-actin is shown (see Supplementary Fig. 5 for corresponding full-length image).

The involvement of the MAPKs JNK1 and JNK2 was investigated by transfection of the cells with siRNAs against JNK-1 and -2. The expression of both isoforms was knocked down together because of the existence of extensive functional redundancy in numerous responses^27^. The expression of JNK1 mRNA was significantly decreased by 50% in vehicle-treated cells (p = 0.007) with a non-significant reduction (31%; p = 0.130) observed in those stimulated with IL-33 (Fig. 3A). Similarly, JNK2 mRNA expression was significantly decreased by 65% and 31% in vehicle- and IL-33-treated cells respectively (p < 0.001 and p = 0.001 respectively) (Fig. 3B). Western blot analysis confirmed the knockdown at the protein level (Fig. 3C). The significant reduction in MCP-1 and ICAM-1 expression by IL-33 observed in cells transfected with the negative control (p = 0.006 and p = 0.009 respectively) was attenuated following knockdown of JNK1/2 (Fig. 3D,E), with a significant increase in expression seen for MCP-1 (p = 0.016), thereby indicating a requirement for this kinase in the action of IL-33.

**Figure 4:**
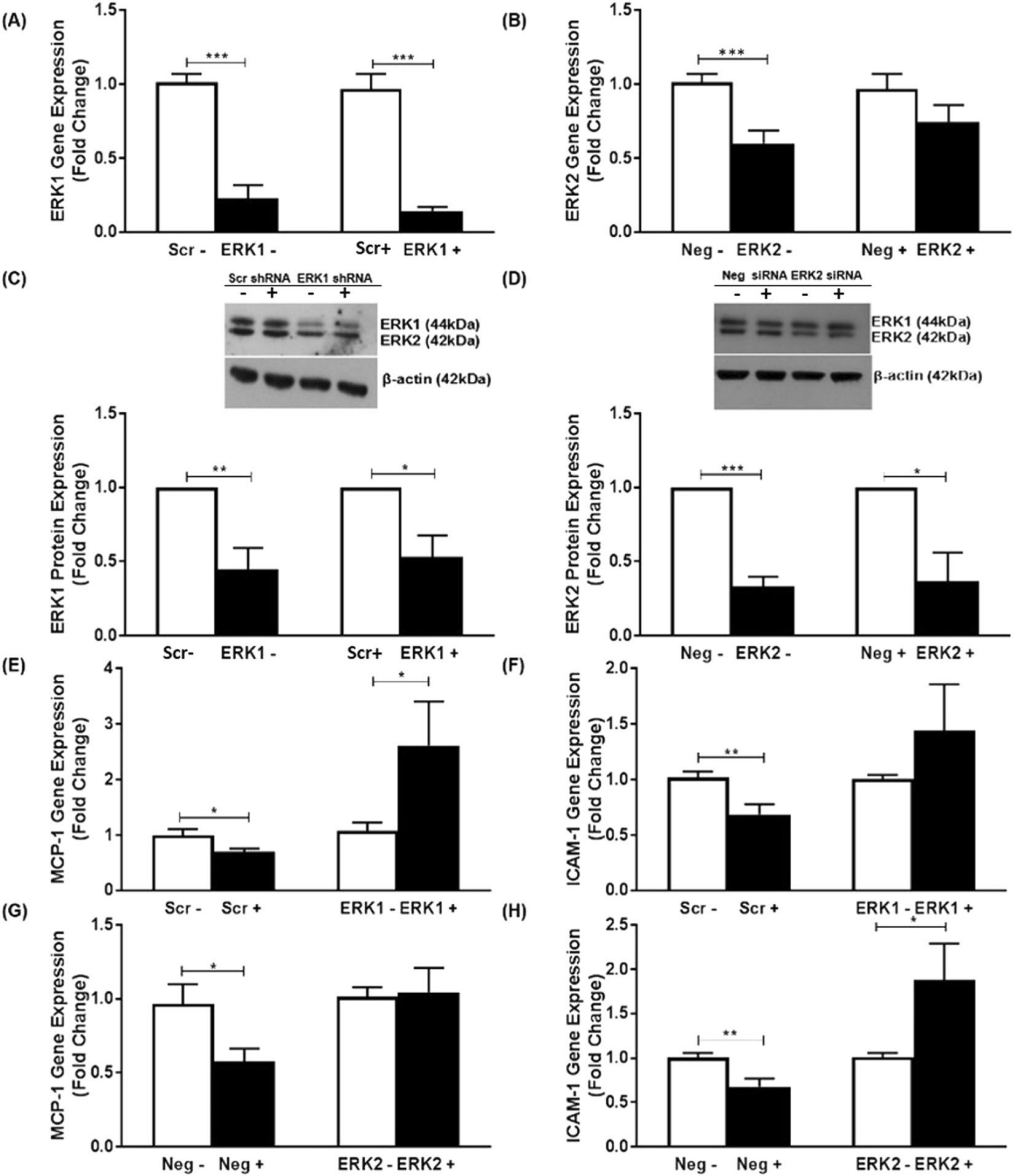
ERK1/2 is required for the IL-33-mediated inhibition of MCP-1 and ICAM-1 expression in human macrophages. Knockdown using adenovirus encoding shRNA against ERK1 or scramble (Scr) sequence or siRNA against ERK2 or negative control sequence (Neg) was carried out as Materials and Methods. THP-1 macrophages were then incubated for 12 h in the presence of vehicle (−) or 25 ng/ml IL-33 (+). Expression of mRNA for ERK1 (**A**), ERK2 (**B**), MCP-1 (**E**,**G**) and ICAM-1 (**F**,**H**) was analyzed by RT-qPCR. Data represents mean ± SEM from three to five independent experiments. The expression of ERK1/2 protein levels was determined by Western blot analysis using β-actin as a control (**C**,**D**). A representative image with signal from immunoreactive ERK1/2 or β-actin is shown (see Supplementary Figs 6 and 7 for corresponding full-length image for panels (**C**,**D**) respectively) with the histogram below it indicating ERK-1 or -2 protein expression normalized to β-actin from three to four independent experiments. The knockdown of ERK1/2 in vehicle- or IL-33 treated cells was determined in relation to the scramble/negative control, which was arbitrarily assigned as 1 (**A**–**D**). The IL-33-mediated changes in MCP-1 and ICAM-1 expression in the scramble/negative control was compared to that following knockdown of ERK1/2 (**E**–**H**) with values from cells treated with scramble/negative control or ERK1 shRNA/ERK2 siRNA and then incubated with vehicle alone given an arbitrary value of 1. Statistical analysis was carried out using an unpaired Student’s t-test (**A**,**E**,**F**) or Man Whitney U test (**B**–**D**,**G**,**H**) (*p ≤ 0.05, **p ≤ 0.01, ***p ≤ 0.001).

For the ERK pathway, the expression was initially knocked down using adenoviral-encoding shRNA against individual isoforms. Because of only a slight reduction of ERK2 at the protein level in IL-33-treated cells (~22%; data not shown), siRNA-mediated knockdown was carried out for this isoform. The data for ERK1 shRNA and ERK2 siRNA are presented in Fig. 4. The knockdown of ERK1 mRNA was 78% and 86% in vehicle- and IL-33-treated cells respectively (p < 0.001 in both cases) and for ERK2 of 40% and 26% respectively (p = 0.001 and 0.168 respectively) (Fig. 4A,B). The knockdown was specific to the isoform (Supplementary Fig. 3A,B). Thus, the expression of ERK1 was not reduced following knockdown of ERK2 and *vice versa* the expression of ERK2 was not decreased following knockdown of ERK1. Western blot analysis revealed that the knockdown of ERK1 protein was 56% and 48% in vehicle- and IL-33-treated cells respectively (p = 0.009 and p = 0.018 respectively) and for ERK2 of 68% and 64% (p = 0.001 and p = 0.031 respectively) (Fig. 4C,D). Thus, a significant reduction of both ERK1 and -2 proteins was obtained in vehicle- and IL-33-treated cells. Analysis of data from multiple experiments revealed that the knockdown was also specific at the protein level (Supplementary Fig. 3C,D). The significant IL-33-mediated decrease in MCP-1 and ICAM-1 mRNA expression (p = 0.031 and p = 0.005 respectively for ERK1 shRNA, and p = 0.021 and p = 0.004 respectively for ERK2 siRNA) was attenuated following knockdown of ERK-1 or -2 (Fig. 4E–H). In the case of ERK1 knockdown, the reduction in MCP-1 expression by IL-33 was reversed with significantly increased levels being observed (Fig. 4E; p = 0.018). Similarly, in the case of ERK2 knockdown, the IL-33-mediated reduction in ICAM-1 expression was reversed with significantly increased levels also being observed (Fig. 4H; p = 0.039).

**Figure 5:**
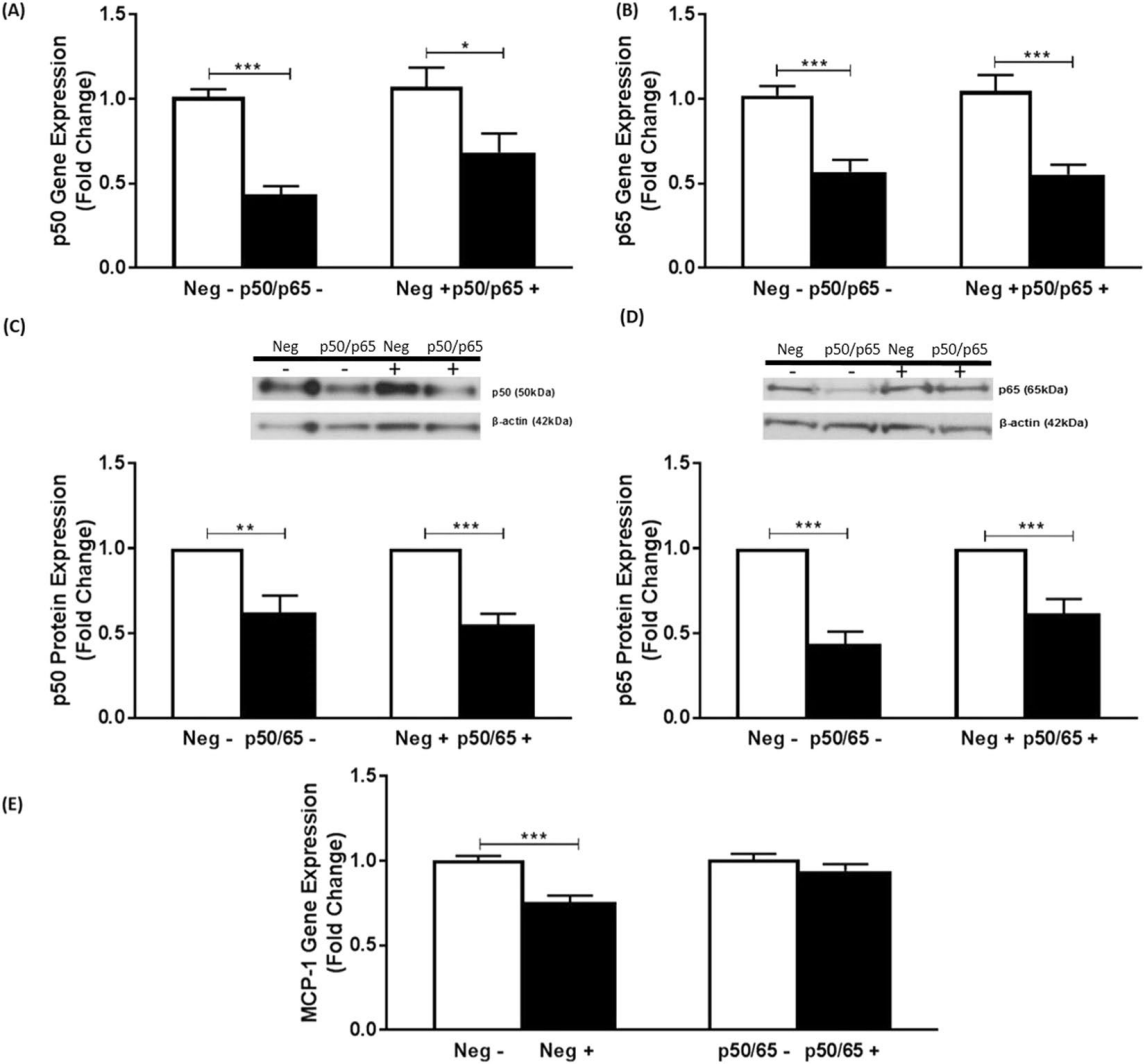
Both p50 and p65 NFκB are required for the IL-33-mediated inhibition of MCP-1 expression in human macrophages. Knockdown using siRNA against p50/p65 NFκB or negative control sequence (Neg) was carried out as Materials and Methods. THP-1 macrophages were then incubated for 12 h in the presence of vehicle (−) or 25 ng/ml IL-33 (+). Expression of mRNA for p50 (**A**) or p65 (**B**) or MCP-1 (**E**) was analyzed by RT-qPCR. Data represents mean ± SEM from three independent experiments. The expression of p50 or p65 protein levels was determined by Western blot analysis using β-actin as a control. A representative image with signal from immunoreactive p50, p65 or β-actin is shown (see Supplementary Figs 8 and 9 for corresponding full-length image for panels (**C**,**D**) respectively) with the histogram below it indicating p50 or p65 protein expression normalized to β-actin. The knockdown of p50/p65 in vehicle- or IL-33 treated cells was determined in relation to the negative control, which was arbitrarily assigned as 1 (**A**–**D**). The IL-33-mediated changes in MCP-1 expression in the negative control was compared to that following knockdown of p50/p65 (**E**) with values from cells transfected with negative control siRNA or p50/p65 siRNA and then treated with vehicle alone given an arbitrary value of 1. Statistical analysis was carried out using an unpaired Student’s t test (*p ≤ 0.05, **p ≤ 0.01, ***p ≤ 0.001).

p50 and p65 are the major NF-κB family members implicated in cytokine signaling and the control of the inflammatory response^2^. Their expression was therefore knocked down using siRNA. In the case of p50, a significant decrease in mRNA expression of 55% and 32% was observed in vehicle- and IL-33-treated cells respectively (p < 0.001 and p = 0.022 respectively) (Fig. 5A). For p65, the decrease was 51% and 45% respectively (p < 0.001 in both cases) (Fig. 5B). Western blot analysis confirmed the knockdown at the protein level (Fig. 5C,D). Thus, the knockdown of p50 was 38% and 45% in vehicle- and IL-33-treated cells respectively (p = 0.003 and p < 0.001 respectively) and 56% and 38% respectively in the case of the p65 isoform (p < 0.001 and p = 0.001 respectively) (Fig. 5C,D). In the case of MCP-1, the significant IL-33-mediated reduction of mRNA expression observed in cells transfected with negative control siRNA (p < 0.001) was attenuated following knockdown of both NF-κB isoforms (Fig. 5D). Conclusions could not be made for ICAM-1 expression as none of the changes were significant, including in cells transfected with negative siRNA (data not shown).

**Figure 6:**
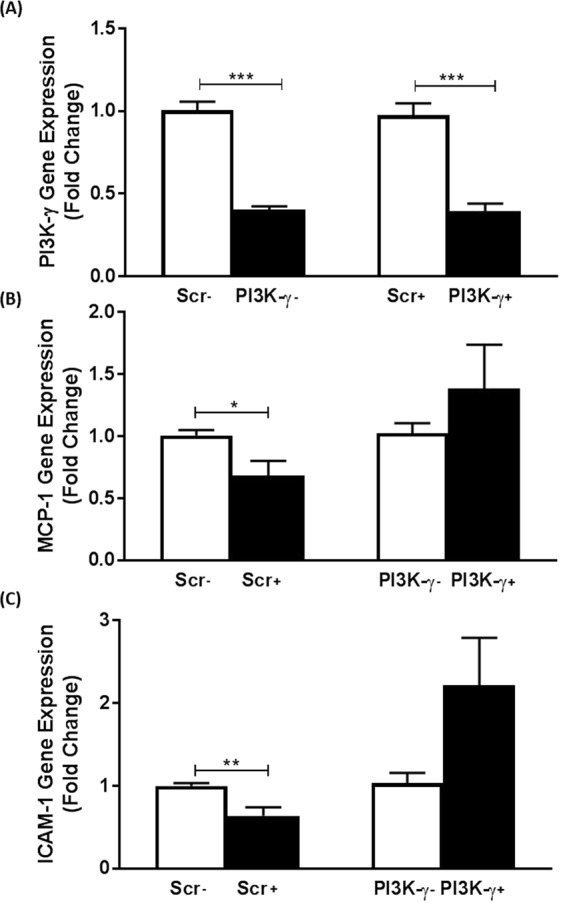
PI3Kγ is required for the IL-33-mediated inhibition of MCP-1 and ICAM-1 expression in human macrophages. Knockdown using adenovirus encoding shRNA against PI3Kγ or scramble (Scr) sequence was carried out as Materials and Methods. THP-1 macrophages were then incubated for 12 h in the presence of vehicle (−) or 25 ng/ml IL-33 (+). Expression of mRNA for PI3Kγ (**A**), MCP-1 (**B**) and ICAM-1 (**C**) was analyzed by RT-qPCR. Data represents mean ± SEM from three to six independent experiments. The knockdown of PI3Kγ in vehicle- or IL-33 treated cells was determined in relation to the scramble control, which was arbitrarily assigned as 1 (**A**). The IL-33-mediated changes in MCP-1 and ICAM-1 expression in the scramble control was compared to that following knockdown of PI3Kγ (**B**,**C**) with values from cells infected with scramble shRNA or PI3Kγ shRNA and then treated with vehicle alone given an arbitrary value of 1. Statistical analysis was carried out using an unpaired Student’s t test (**A**,**C**) or Mann Whitney U test (**B**) (*p ≤ 0.05, **p ≤ 0.01, ***p ≤ 0.001).

The PI3K family member, PI3K-γ, plays an important role in atherosclerosis^2^. Its involvement was therefore investigated by knockdown using adenoviral-encoding shRNA. The knockdown of PI3K-γ mRNA was 60% and 61% respectively in vehicle- and IL-33 treated cells respectively (p < 0.001 in both cases) (Fig. 6A). The significant reduction in MCP-1 and ICAM-1 expression by IL-33 in cells infected with adenovirus encoding scramble shRNA (p = 0.019 and p = 0.002 respectively) was attenuated following knockdown of PI3K-γ (Fig. 6B,C).

## Discussion

IL-33 is a more recently identified IL-1 family member with important functions in regulating infection, inflammation and cancer^21^. The actions of this cytokine in such disorders are mediated via a range of immune cells, including macrophages^21^. We have previously shown inhibition of macrophage foam cell formation *in vitro* and *in vivo* by IL-33^10^. In addition, we demonstrated the requirement of the ST2 receptor for its effect on cholesterol homeostasis and the regulation of gene expression in macrophages *in vitro*^10^. We show here that IL-33 attenuates the expression of MCP-1 and ICAM-1, two major pro-atherogenic genes, in human macrophages and identify the roles of key signaling pathways activated by this cytokine in such regulation using knockdown assays.

An important role for IL-33 in atherosclerosis was identified by studies in the ApoE deficient mouse model system^9^. The disease was attenuated by injection of IL-33 and exacerbated with the soluble ST2 receptor that prevents the cytokine from initiating cellular responses^9^. IL-33 increased the levels of several anti-atherogenic cytokines such as IL-13, decreased the expression of some pro-atherogenic cytokines (e.g. interferon-γ), caused a Th1 to Th2 shift and increased the concentration of anti-oxLDL antibodies^9^. However, a recent study in ApoE^−/−^ mice deficient in either IL-33 or its ST2 receptor failed to find any effect on atherosclerosis^28^. The precise reasons for the discrepancy are currently unclear but differences in the experimental design such as cholesterol levels in the high fat diet and duration of the feeding may have contributed^28^. In addition, not all the actions of IL-33 are anti-atherogenic; for example, Demyanets *et al*.^29^ first showed that IL-33 activates human endothelial cells and increases the expression of MCP-1 and adhesion molecules in these cells *in vitro* and in human atherosclerotic plaques *ex vivo*. Subsequently, Pollheimer *et al*.^30^ also demonstrated that the cytokine causes endothelial cell activation with stronger responses in nonquiescent cells.

Our studies reveal an anti-atherogenic role for IL-33, provide mechanistic insights into such an action and add to the beneficial effects reported in the prevention of obesity^31^. For example, IL-33 decreased the formation of macrophage foam cells *in vitro* and *in vivo*^10^. The cytokine attenuated the expression of several key genes involved in modified LDL uptake and intracellular cholesterol storage and simultaneously induced the expression of genes required for the intracellular transport of this sterol and its efflux out of foam cells^10^. We have also shown that the expression of a disintegrin and metalloproteinase with thrombospondin motifs-4 in human macrophages was inhibited by IL-33^13^, and recently it has been demonstrated that its deficiency in ApoE^−/−^ mice attenuates atherosclerosis development and improves plaque stability^32^. The studies presented here show that IL-33 inhibits the expression of MCP-1 and ICAM-1 that have been demonstrated to play pro-atherogenic roles in mouse model systems^2,8^. Interestingly, IL-33 induces the expression of some of these genes in other cell types; for example, ICAM-1 in human eosinophils^33^ and MCP-1 and ICAM-1 in human endothelial cells^29,30^. This implicates cell-type-specific actions of the cytokine that needs to be investigated further. However, the mechanisms are likely to be complex given that the promoter regions of MCP-1 and ICAM-1 genes contain binding sites for several transcription factors (e.g. signal transducer and activator of transcription-1, -3, and -5; E26 transformation-specific; CCAAT/enhancer binding proteins; activator protein-1; specificity protein-1; farnesoid X receptor; peroxisome proliferator-activated receptors etc), many of which have been shown to be functionally important^34–43^. All these transcription factors also belong to large families, with both activators and repressors, and additional complexity created by large numbers of post-translational modifications, interactions between them or even with other proteins, and epigenetic regulation^36,38–40^. For example, NF-κB, which is known to stimulate MCP-1 and ICAM-1 expression^29,36,40^, consists of five members that can also inhibit gene transcription with additional complexity created by interactions with numerous other transcription factors/proteins, post-translational modifications and epigenetic regulation that also produces suppression of gene expression^44–52^. It is therefore not surprising that experiments in mouse models have not always demonstrated pro-atherogenic roles for the different members^2^.

IL-33 is known to activate several signal transduction pathways such as NF-κB, MAPK and PI3K^21^. Many studies have determined the signaling pathways underlying IL-33 actions with some cell- and gene-specific responses being identified^16–20,53^. For example, IL-33 activates p38 MAPK in lung endothelial cells but not in epithelial cells^20^. The majority of previous research has studied the IL-33-mediated stimulation of gene expression or cellular responses. For instance, IL-33 has been shown to promote ovarian cancer cell growth and metastasis via ERK and JNK signaling pathways^54^. Unfortunately, the signaling pathways underlying the inhibitory actions of IL-33 are not well understood. Such inhibitory actions extend to key processes such as macrophage foam cell formation and immune cell recruitment to atherosclerotic plaques^9,10^, decidual natural killer cell cytotoxicity in early human pregnancy^55^ and cardiac remodelling following myocardial infarction^56^. Using knockdown assays, we show here a requirement of NF-κB, PI3Kγ and MAPK cascades in the IL-33-mediated inhibition of two key pro-atherogenic genes, MCP-1 and ICAM-1 (Figs 2–6). Interestingly, these pathways are required for both the induction and suppression of gene expression by IL-33. Future studies should investigate how differential responses following activation of these signaling pathways are achieved. These are beyond the scope of current studies because of the immense complexity of IL-33 signaling. For example, data mining has revealed an integrated pathway map of IL-33 and its receptor that consists of 681 proteins and 765 reactions^57^. The complexity can be gauged by the involvement of 9 transcriptional regulators, 2492 gene regulation events and 740 enzyme catalysis events^57^. In addition, quantitative phosphoproteomic analysis has revealed IL-33-mediated changes in phosphorylation at 1050 sites in 672 proteins^58^. Activation of numerous such proteins together with protein-protein interactions may make a key contribution to such differential effects.

In conclusion, the studies presented here supports that IL-33 exerts anti-atherogenic actions. The data demonstrates that the cytokine causes a novel decrease in the expression of MCP-1 and ICAM-1. In addition, key roles for ERK1/2, p38α, JNK1/2, NF-κB and PI3Kγ were identified in such an inhibitory action of the cytokine.

## Materials and Methods

### Reagents

Human THP-1 cell line was from Sigma-Aldrich and recombinant human IL-33 was supplied by Peprotech. Antibodies were from Cell Signaling Technology [anti-p44/p42 (ERK1/2) (9102), anti-p38 (9212), anti-JNK1/2 (9252), NF-κB p105/p50 (3035)], Santa Cruz Biotechnology [NF-κB p65 (sc-372), β-Actin (sc-130656)] or Sigma-Aldrich [β-Actin (A2228)].

### Cell culture

HMDMs were isolated from buffy coats supplied by the National Blood Service Wales, which were processed immediately following collection using the Ficoll-Hypaque purification method as previously described^10,23,25^. Informed consent for each donor was granted to the Welsh Blood Service for the use of human blood for non-transfusion purposes^10,23,25^. All methods were carried out in accordance with the relevant guidelines and regulation (all experimental protocols were approved by the School of Biosciences and Cardiff University)^10,23,25^. THP-1 and HMDM were cultured in RPMI1640 medium with stable glutamine containing 10% (v/v) heat-inactivated foetal calf serum, penicillin (100 U/ml) and streptomycin (100 µg/ml) at 37 °C in a humidified atmosphere containing 5% (v/v) CO_2_^6,13,23–25^. THP-1 monocytes were differentiated into macrophages by incubation for 24 h with 0.16 µM of phorbol 12-myristate 13-acetate (PMA)^6,13,23–25^.

### Real-time quantitative PCR (RT-qPCR)

Isolation of RNA, reverse transcription and RT-qPCR of the resulting cDNA were carried out as described elsewhere^6,10,25^. The sequences of the primers used are given in Supplementary Table 1. The comparative ΔΔC_t_ method was used to represent relative expression normalized to the housekeeping gene glyceraldehyde 3-phosphate dehydrogenase (GAPDH)^10,25^.

### Western blotting

Size-fractionation of equal amounts of protein was carried out by SDS-PAGE alongside comparative molecular weight size markers and subjected to Western blot analysis as described elsewhere^6,10,23,25^.

### RNA interference

Knockdown assays were performed by transfections using validated siRNAs targeting human ERK2 (SI00300755), JNK1 (SI02758637), JNK2 (SI02222920), p50 (SI02654932) and p65 (SI00301672) from Qiagen. Negative control siRNA (AM4611) was purchased from Invitrogen. Stock solutions of lyophilized siRNA were prepared in nuclease-free water in accordance with the manufacturer’s instructions (Qiagen and Invitrogen).

siRNA transfections were performed in THP-1 monocytes before the addition of PMA for differentiation into macrophages in accordance to the manufacturer’s instructions (Polyplus Transfection) as described in previous studies^6,13,25^. The preparation of small hairpin RNA (shRNA)-encoding recombinant adenovirus that target ERK-1, ERK-2, p38α or PI3Kγ has been previously described^13,24^. The adenovirus was then added at a multiplicity of infection of 100 based on previous optimisation studies^24,25^. The cells were left for 2.5 hours at 37 °C in a humidified incubator containing 5% (v/v) CO_2_ before the addition of 0.16 µM PMA. Incubation of the cells at 37 °C in a humidified incubator containing 5% (v/v) CO_2_ was carried out for 48 hours before stimulation with IL-33 or vehicle for the requisite time. Following cytokine stimulation, RNA or protein was isolated as required.

### Statistical analysis

The data are presented as mean ± SEM with normality determined using the Shapiro-Wilk test. Single comparisons were performed using an unpaired Student’s *t* test, or if the data were not normally distributed using a Mann Whitney U test. The results were regarded as significant where *p ≤ 0.05, **p ≤ 0.01 and ***p ≤ 0.001.

## Supplementary information

Supplementary Data

## Data Availability

The datasets produced and/or analyzed during this study are available from the corresponding author on reasonable request.

## References

[1] McLaren JE, Michael DR, Ashlin TG, Ramji DP (2011). Cytokines, macrophage lipid metabolism and foam cells: Implications for cardiovascular disease therapy. Prog Lipid Res.

[2] Buckley ML, Ramji DP (2015). The influence of dysfunctional signaling and lipid homeostasis in mediating the inflammatory responses during atherosclerosis. Biochim Biophys Acta.

[3] Moore KJ, Sheedy FJ, Fisher EA (2013). Macrophages in atherosclerosis: a dynamic balance. Nat Rev Immunol.

[4] Michael DR, Ashlin TG, Buckley ML, Ramji DP (2012). Macrophages, lipid metabolism and gene expression in atherogenesis: a therapeutic target of the future?. Clin Lipidol.

[5] Michael DR (2013). Differential regulation of macropinocytosis in macrophages by cytokines: Implications for foam cell formation and atherosclerosis. Cytokine.

[6] Salter RC (2016). The role of mitogen-activated protein kinases and sterol receptor coactivator-1 in TGF-β-regulated expression of genes implicated in macrophage cholesterol uptake. Sci Rep.

[7] Moss JWE, Ramji DP (2016). Cytokines: roles in atherosclerosis disease progression and potential therapeutic targets. Future Med Chem.

[8] Ramji DP, Davies TS (2015). Cytokines in atherosclerosis: Key players in all stages of disease and promising therapeutic targets. Cytokine Growth Factor Rev.

[9] Miller AM (2008). IL-33 reduces the development of atherosclerosis. J Exp Med.

[10] McLaren JE (2010). IL-33 reduces macrophage foam cell formation. J Immunol.

[11] Zhang HF (2017). IL-33 promotes IL-10 production in macrophages: a role for IL-33 in macrophage foam cell formation. Exp Mol Med.

[12] Joshi AD (2010). Interleukin-33 contributes to both M1 and M2 chemokine marker expression in human macrophages. BMC Immunol.

[13] Ashlin TG (2014). The anti-atherogenic cytokine interleukin-33 inhibits the expression of a disintegrin and metalloproteinase with thrombospondin motifs-1, -4 and -5 in human macrophages: Requirement of extracellular signal-regulated kinase, c-Jun N-terminal kinase and phosphoinositide 3-kinase signaling pathways. Int J Biochem Cell Biol.

[14] Schmitz J (2005). IL-33, an interleukin-1-like cytokine that signals via the IL-1 receptor-related protein ST2 and induces T helper type 2-associated cytokines. Immunity.

[15] Kakkar R, Lee RT (2008). The IL-33/ST2 pathway: therapeutic target and novel biomarker. Nat Rev Drug Discov.

[16] Brint EK (2002). Characterization of signaling pathways activated by the interleukin 1 (IL-1) receptor homologue T1/ST2. A role for Jun N-terminal kinase in IL-4 induction. J Biol Chem.

[17] Funakoshi-Tago M (2008). TRAF6 is a critical signal transducer in IL-33 signaling pathway. Cell Signal.

[18] Funakoshi-Tago M, Tago K, Sato Y, Tominaga S, Kasahara T (2011). JAK2 is an important signal transducer in IL-33-induced NF-κB activation. Cell Signal.

[19] Choi YS (2009). Interleukin-33 induces angiogenesis and vascular permeability through ST2/TRAF6-mediated endothelial nitric oxide production. Blood.

[20] Yagami A (2010). IL-33 mediates inflammatory responses in human lung tissue cells. J Immunol.

[21] Liew FY, Girard JP, Turnquist HR (2016). Interleukin-33 in health and disease. Nat Rev Immunol.

[22] Qin Z (2012). The use of THP-1 cells as a model for mimicking the function and regulation of monocytes and macrophages in the vasculature. Atherosclerosis.

[23] Gallagher H (2019). Dihomo-γ-linolenic acid inhibits several key cellular processes associated with atherosclerosis. Biochim Biophys Acta.

[24] Li N (2010). ERK is integral to the IFN-gamma-mediated activation of STAT1, the expression of key genes implicated in atherosclerosis, and the uptake of modified lipoproteins by human macrophages. J Immunol.

[25] Michael DR, Salter RC, Ramji DP (2012). TGF-beta inhibits the uptake of modified low density lipoprotein by human macrophages through a Smad-dependent pathway: A dominant role for Smad-2. Biochim Biophys Acta.

[26] Mead JR, Ramji DP (2002). The pivotal role of lipoprotein lipase in atherosclerosis. Cardiovasc Res.

[27] Saba-El-Leil MK, Frémin C, Meloche S (2016). Redundancy in the world of MAP kinases: All for one. Front Cell Dev Biol.

[28] Martin P (2015). Atherosclerosis severity is not affected by a deficiency in IL-33/ST2 signaling. Immun Inflamm Dis.

[29] Demyanets S (2011). Interleukin-33 induces expression of adhesion molecules and inflammatory activation in human endothelial cells and in human atherosclerotic plaques. Arterioscler Thromb Vasc Biol.

[30] Pollheimer J (2013). Interleukin-33 drives a proinflammatory endothelial activation that selectively targets nonquiescent cells. Arterioscler Thromb Vasc Biol.

[31] Miller AM (2010). Interleukin-33 induces protective effects in adipose tissue inflammation during obesity in mice. Circ Res.

[32] Kumar S (2016). Loss of ADAMTS4 reduces high fat diet-induced atherosclerosis and enhances plaque stability in ApoE(−/−) mice. Sci Rep.

[33] Chow JY, Wong CK, Cheung PF, Lam CW (2010). Intracellular signaling mechanisms regulating the activation of human eosinophils by the novel Th2 cytokine IL-33: implications for allergic inflammation. Cell Mol Immunol.

[34] Walter MJ, Look DC, Tidwell RM, Roswit WT, Holtzman MJ (1997). Targeted inhibition of interferon-gamma-dependent intercellular adhesion molecule-1 (ICAM-1) expression using dominant-negative Stat1. J Biol Chem.

[35] de Launoit Y, Audette M, Pelczar H, Plaza S, Baert JL (1998). The transcription of the intercellular adhesion molecule-1 is regulated by Ets transcription factors. Oncogene.

[36] Lee SJ, Hou J, Benveniste EN (1998). Transcriptional regulation of intercellular adhesion molecule-1 in astrocytes involves NF-kappaB and C/EBP isoforms. J Neuroimmunol.

[37] Qin P, Borges-Marcucci LA, Evans MJ, Harnish DC (2005). Bile acid signaling through FXR induces intracellular adhesion molecule-1 expression in mouse liver and human hepatocytes. Am J Physiol Gastrointest Liver Physiol.

[38] Yang XP (2005). Signal transducer and activator of transcription 3alpha and specificity protein 1 interact to upregulate intercellular adhesion molecule-1 in ischemic-reperfused myocardium and vascular endothelium. Arterioscler Thromb Vasc Biol.

[39] Zhang Y, Qu Y, Niu T, Wang H, Liu K (2017). O-GlcNAc modification of Sp1 mediates hyperglycaemia-induced ICAM-1 up-regulation in endothelial cells. Biochem Biophys Res Commun.

[40] Ueda A (1994). NF-kappa B and Sp1 regulate transcription of the human monocyte chemoattractant protein-1 gene. J Immunol.

[41] Marini E (2008). HIV-1 matrix protein p17 binds to monocytes and selectively stimulates MCP-1 secretion: role of transcriptional factor AP-1. Cell Microbiol 10, 655-666.

[42] Tanimoto A (2008). Monocyte chemoattractant protein-1 expression is enhanced by granulocyte-macrophage colony-stimulating factor via Jak2-Stat5 signaling and inhibited by atorvastatin in human monocytic U937 cells. J Biol Chem.

[43] Naidenow J (2016). Peroxisome proliferator-activated receptor (PPAR) α and δ activators induce ICAM-1 expression in quiescent non stimulated endothelial cells. J Inflamm (Lond).

[44] Yu X (2016). p65 down-regulates DEPTOR expression in response to LPS stimulation in hepatocytes. Gene.

[45] Campbell KJ, Rocha S, Perkins ND (2004). Active repression of antiapoptotic gene expression by RelA(p65) NF-kappa B. Mol Cell.

[46] Lin YC, Hsu EC, Ting LP (2009). Repression of hepatitis B viral gene expression by transcription factor nuclear factor-kappaB. Cell Microbiol.

[47] O’Hara SP (2010). NFkappaB p50-CCAAT/enhancer-binding protein beta (C/EBPbeta)-mediated transcriptional repression of microRNA let-7i following microbial infection. J Biol Chem.

[48] Janbandhu VC, Singh AK, Mukherji A, Kumar V (2010). p65 negatively regulates transcription of the cyclin E gene. J Biol Chem.

[49] Elsharkawy AM (2010). The NF-kappaB p50:p50:HDAC-1 repressor complex orchestrates transcriptional inhibition of multiple pro-inflammatory genes. J Hepatol.

[50] Leidner J (2014). SUMOylation attenuates the transcriptional activity of the NF-κB subunit RelB. J Cell Biochem.

[51] Rippe RA, Schrum LW, Stefanovic B, Solís-Herruzo JA, Brenner DA (1999). NF-kappaB inhibits expression of the alpha1(I) collagen gene. DNA Cell Biol.

[52] Dong J, Jimi E, Zhong H, Hayden MS, Ghosh S (2008). Repression of gene expression by unphosphorylated NF-kappaB p65 through epigenetic mechanisms. Genes Dev.

[53] Kamekura R (2012). The role of IL-33 and its receptor ST2 in human nasal epithelium with allergic rhinitis. Clin Exp Allergy.

[54] Tong X (2016). Interleukin-33 predicts poor prognosis and promotes ovarian cancer cell growth and metastasis through regulating ERK and JNK signaling pathways. Mol Oncol.

[55] Hu WT (2015). Decidual stromal cell-derived IL-33 contributes to Th2 bias and inhibits decidual NK cell cytotoxicity through NF-κB signaling in human early pregnancy. J Reprod Immunol.

[56] Yin H (2014). IL-33 attenuates cardiac remodeling following myocardial infarction via inhibition of the p38 MAPK and NF-κB pathways. Mol Med Rep.

[57] Pinto SM (2018). A network map of IL-33 signaling pathway. J Cell Commun Signal.

[58] Pinto SM (2015). Quantitative phosphoproteomic analysis of IL-33-mediated signaling. Proteomics.

