# Supplementary material for: The interleukin-33-mediated inhibition of expression of two key genes implicated in atherosclerosis in human macrophages requires MAP kinase, phosphoinositide 3-kinase and nuclear factor-κB signaling pathways
Source: Sci Rep. 2019 Aug 5;9:11317. doi: 10.1038/s41598-019-47620-8 (PMC6683160; doi:10.1038/s41598-019-47620-8)
Supplement: Supplementary file 1 — Supplementary Data [file 41598_2019_47620_MOESM1_ESM.pdf]

The interleukin-33-mediated inhibition of expression of two key genes implicated in atherosclerosis in human macrophages requires MAP kinase, phosphoinositide 3-kinase and nuclear factor-κB signaling pathways

Melanie L. Buckley[1], Jessica O. Williams[1], Yee-Hung Chan[1], Lucia Laubertová[1,2], Hayley Gallagher[1], Joe W. E. Moss[1] and Dipak P. Ramji[1*]

[1]: Cardiff School of Biosciences, Cardiff University, Sir Martin Evans Building, Museum Avenue, Cardiff CF10 3AX, United Kingdom

[2]: Institute of Medical Chemistry, Biochemistry and Clinical Biochemistry, Faculty of Medicine, Comenius University, Sasinkova 2, 813 72, Bratislava, Slovakia

* Corresponding author: Ramji@Cardiff.ac.uk

Supplementary Table 1. Primers used for RT-qPCR.

Human Gene	Forward Primer (5' to 3')	Reverse Primer (5' to 3')
**ERK1**	GCAGGACCTGATGGAGACTGAC	CCAGAATGCAGCCCACAGAC
**ERK2**	GCGCTACACCAACCTCTCGT	CACGGTGCAGAACGTTAGCTG
**GAPDH**	GAAGGTGAAGGTCGGAGTC	GAAGATGGTGATGGGATTTC
**ICAM-1**	ACGCTGAGCTCCTCTGCTACTC	GGGCAGGATGACTTTTGAGG
**JNK1**	TCTGGTATGATCCTTCTGAAGCA	TCCTCCAAGTCCATAACTTCCTT
**JNK2**	GAAACTAAGCCGTCCTTTTCAGA	TCCAGCTCCATGTGAATAACCT
**MCP-1**	CATTGTGGCCAAGGAGATCTG	CTTCGGAGTTTGGGTTTGCTT
**p38α**	GTGGTACAGGGCTCCTGAGA	TATGCATCCCACTGACCAAA
**PI3Kγ**	TCTGATGGATATTCCCGAAAGCC	CTCACCCACTGGAAGTTTTTGAT

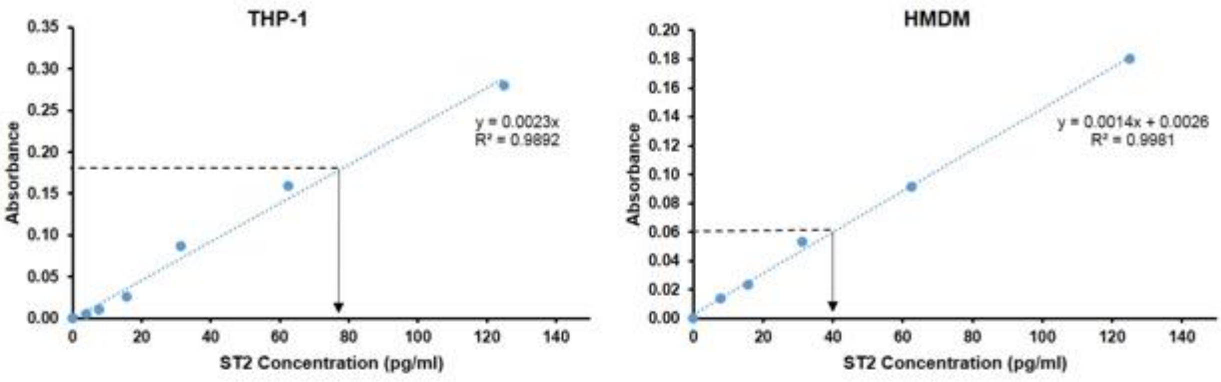

Supplementary Figure 1. ST2 receptor is expressed by both THP-1 macrophages and HMDM

ELISA were carried out on different concentrations of standards and cell lysates (100 μl from 2 x 10^6 THP-1 macrophages and 6 x 10^6 HMDM) using Human ST2/IL-33 R DuoSet ELISA (R & D Systems). The standard curves from representative experiments used to determine the expression of the ST2 receptor are shown. The values for ST2 levels from three independent experiments were 77.6 ± 3.2 pg/ml for THP-1 macrophages and 39.8 ± 1.0 pg/ml for HMDM.

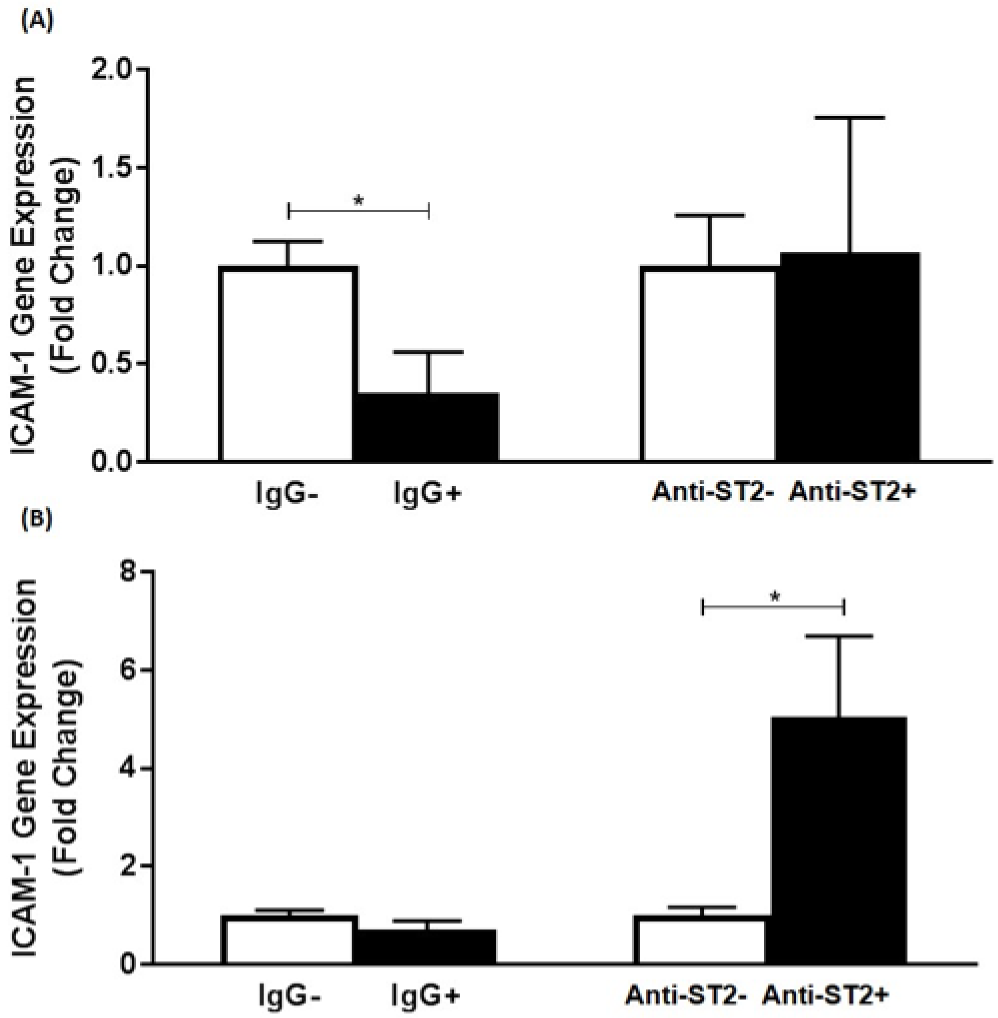

Supplementary Figure 2. The IL-33-mediated suppression of ICAM-1 expression is attenuated by polyclonal antibody to the ST2 receptor

THP-1 macrophages (A) or HMDM (B) were pre-treated for 1 h with 10 μg/ml human ST2/IL-33 R antibody (AF523; R & D Systems) or isotype control antibody (ab97221; Abcam) followed by 12 h in the presence of vehicle (-) or 25 ng/ml IL-33 (+). The expression of ICAM-1 was determined by RT-qPCR and normalized to GAPDH. In each case, the expression of ICAM-1 in cells treated with vehicle has been arbitrarily assigned as 1. Data represents mean ± SEM from 4-6 (A) or 7-10 (B) independent experiments. Statistical analysis was carried out using an unpaired Student's t-test (*$p \leq 0.05$).

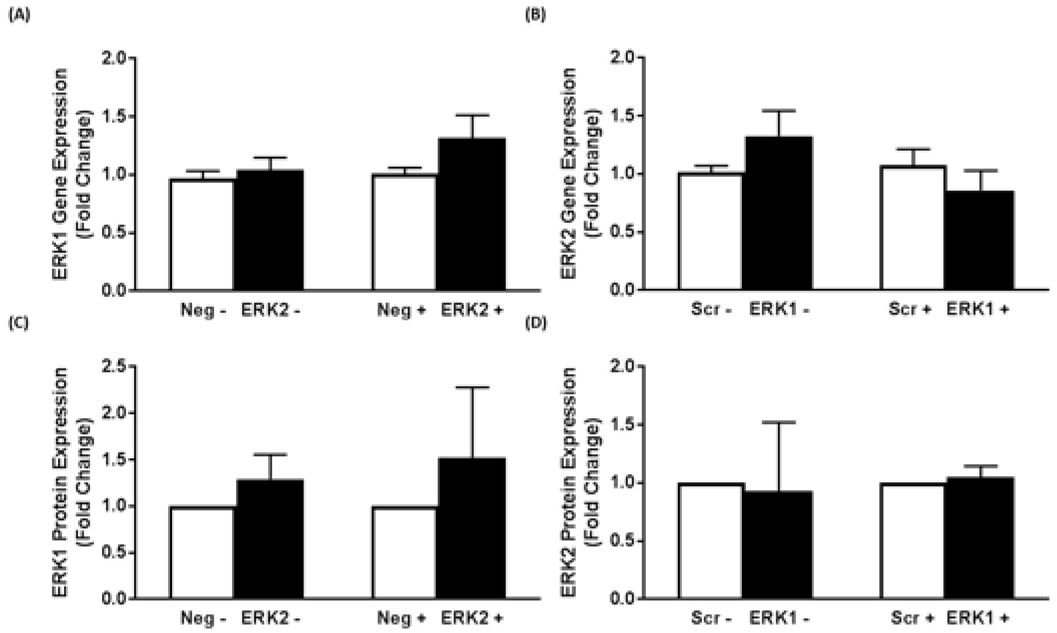

Supplementary Figure 3. Specificity of knockdown assays

RNA and proteins from studies shown in Figure 4 were used. Expression of mRNA for ERK1 (A) or ERK2 (B) was analyzed by RT-qPCR. Data represents mean ± SEM from three to six independent experiments. The expression of ERK1 (C) and ERK2 (D) protein levels were normalized to β-actin from three to four independent experiments. The values from control samples [Scr or Neg in the presence of vehicle (-) or IL-33 (+)] were arbitrarily assigned as 1. Statistical analysis was carried out using an unpaired Student's t-test (none of the changes in expression were significant).

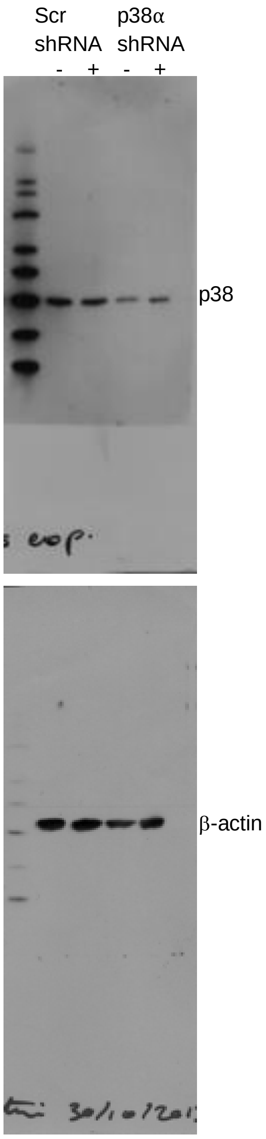

Supplementary Figure 4. Full length Western blots corresponding to Fig. 2B

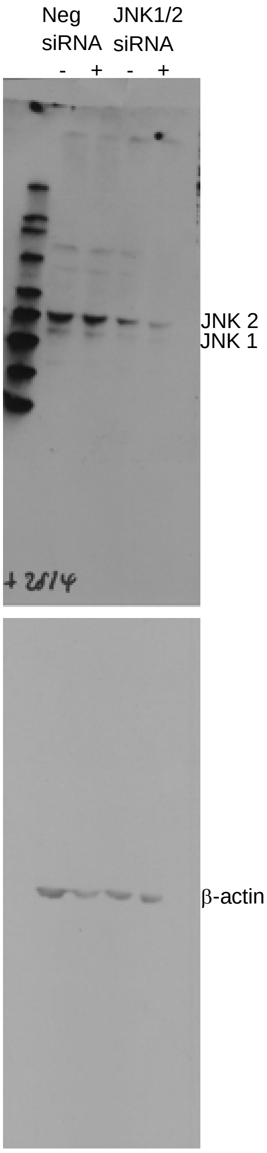

Supplementary Figure 5. Full length Western blots corresponding to Fig. 3C

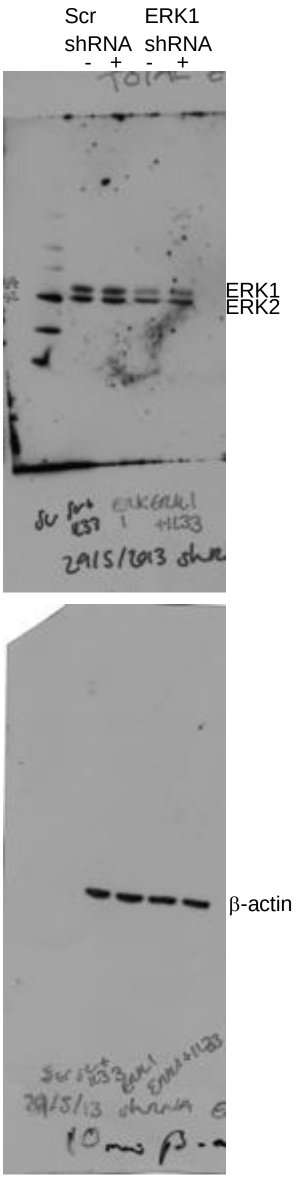

Supplementary Figure 6. Full length Western blots corresponding to Fig. 4C

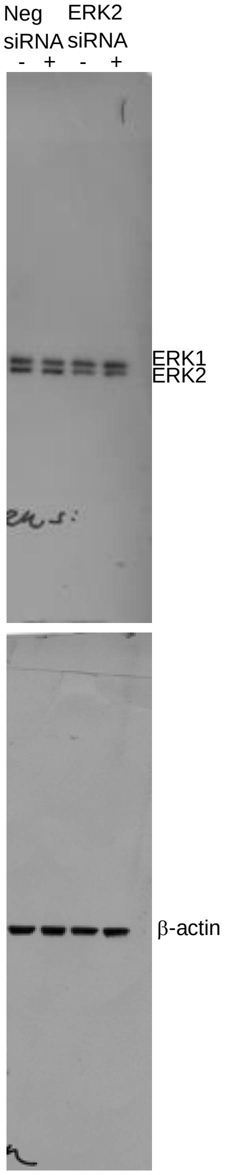

Supplementary Figure 7. Full length Western blots corresponding to Fig. 4D

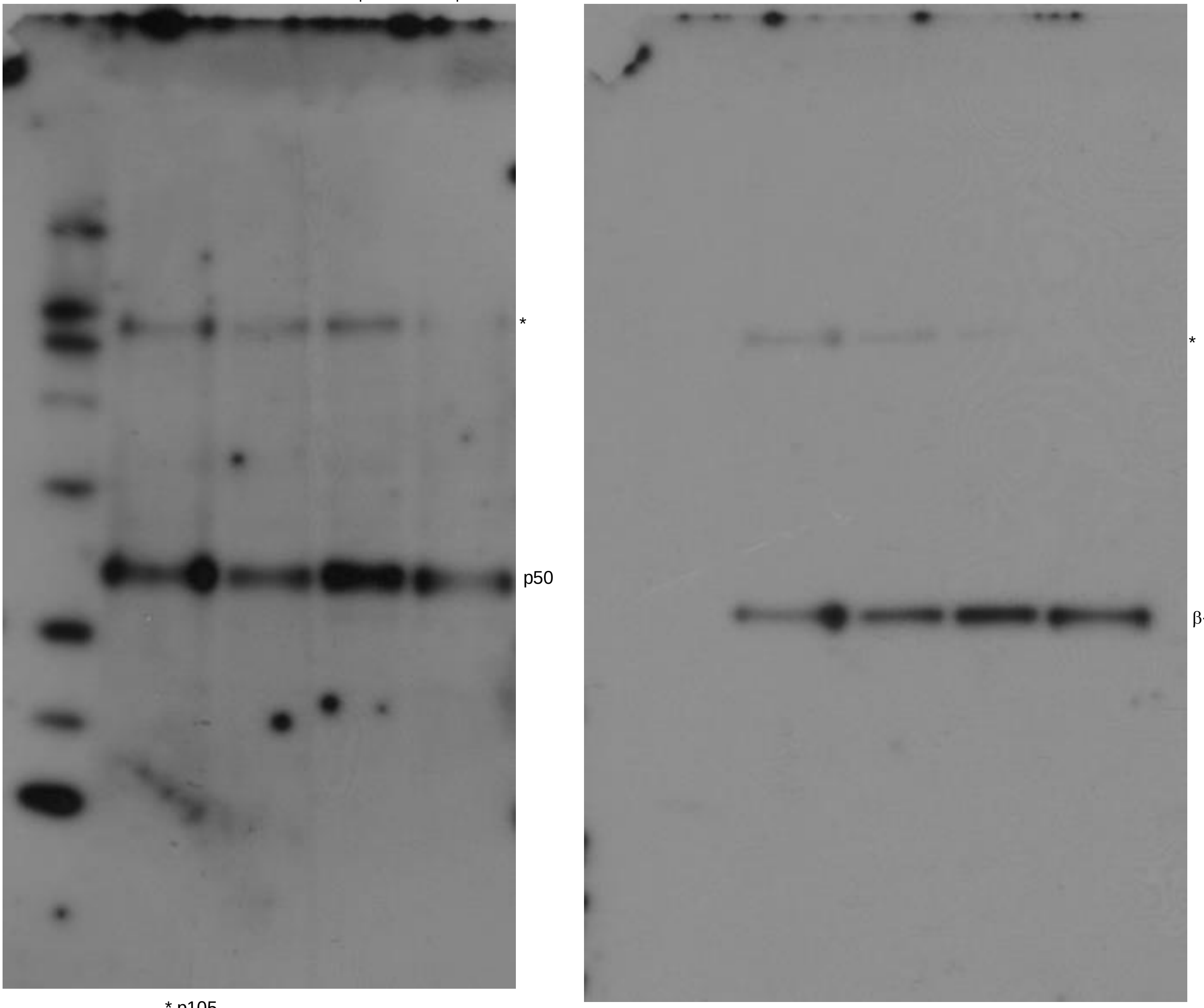

Supplementary Figure 8. Full length Western blots corresponding to Fig. 5C

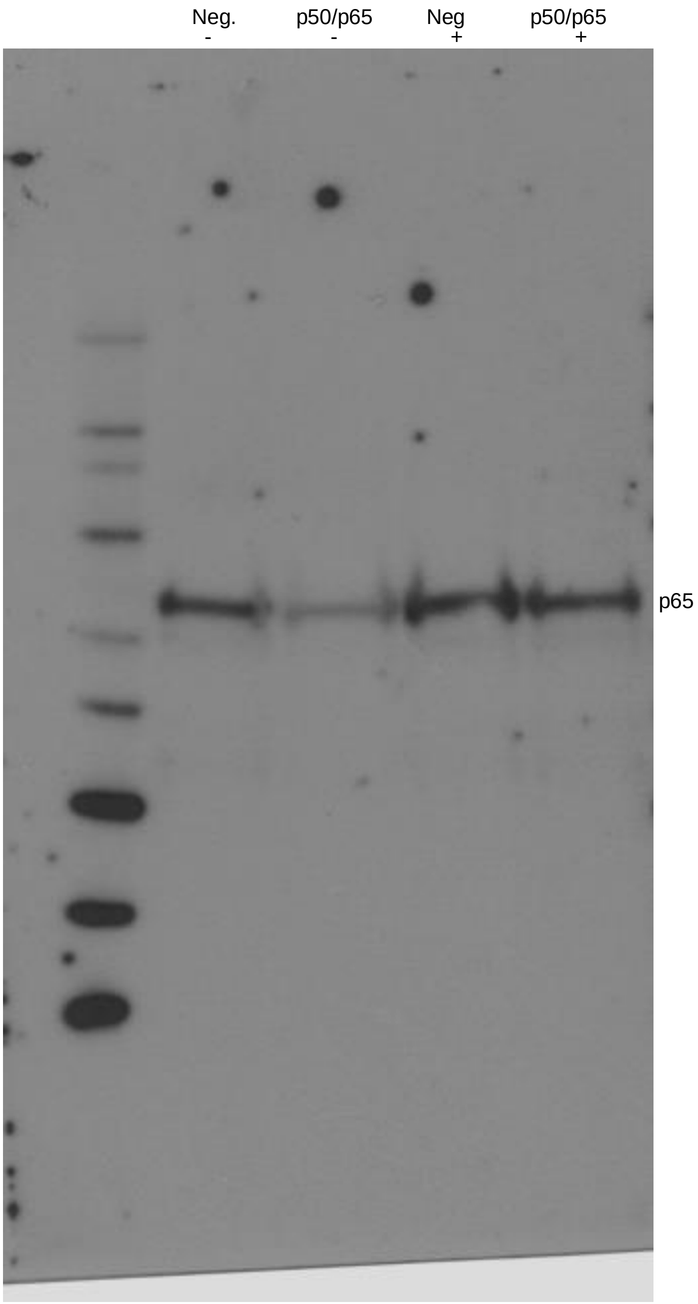

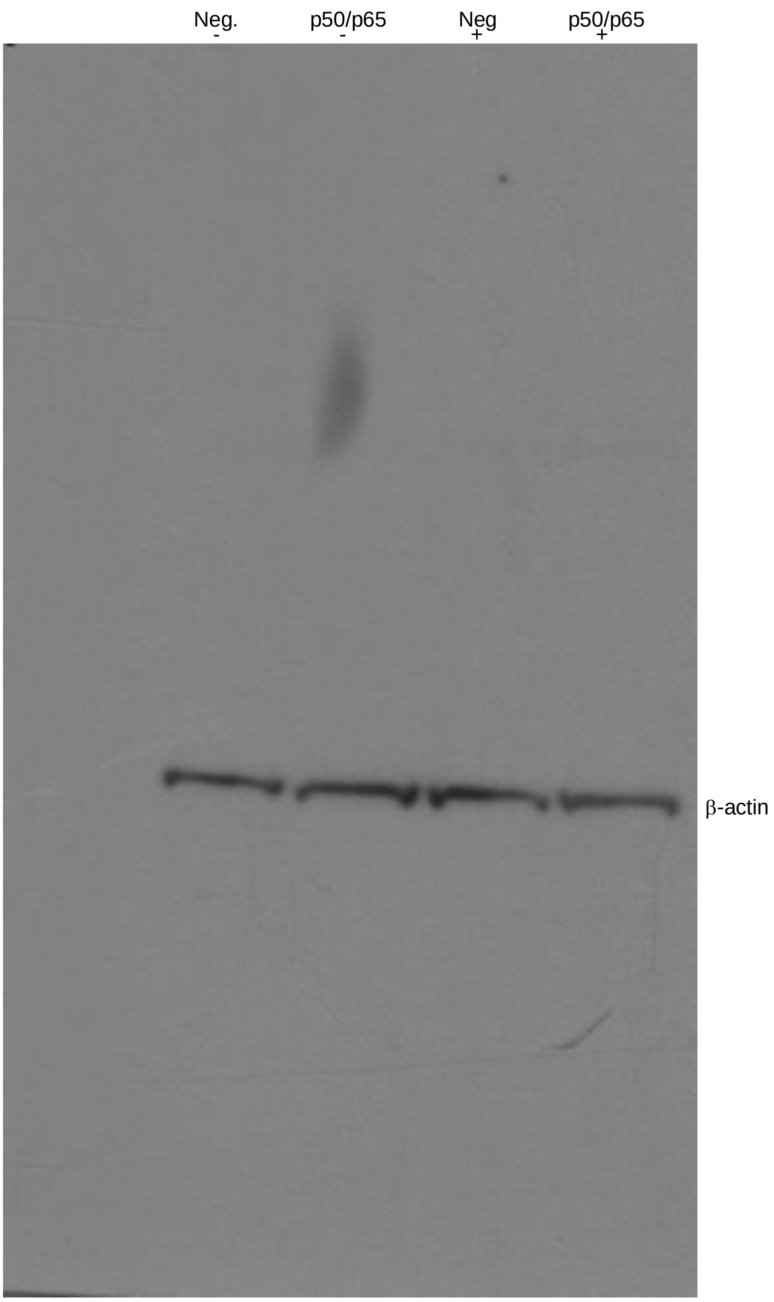

Supplementary Figure 9. Full length Western blots corresponding to Fig. 5D
